# Sjogren’s syndrome is associated with higher rate of non-home discharge after primary hip arthroplasty and higher transfusion rates after primary hip or knee arthroplasty: a U.S. cohort study

**DOI:** 10.1186/s12891-020-03514-9

**Published:** 2020-07-25

**Authors:** Jasvinder A. Singh, John D. Cleveland

**Affiliations:** 1 Medicine Service, VA Medical Center, 700 19th St S, Birmingham, AL 35233 USA; 2 Department of Medicine at School of Medicine, University of Alabama at Birmingham, 510 20th Street South, FOT 805B, Birmingham, AL 35294 United States; 3 Division of Epidemiology at School of Public Health, University of Alabama at Birmingham, 1720 Second Ave South, Birmingham, AL 35294-0022 USA

**Keywords:** Sjogren’s syndrome, Epidemiology, Transfusion, Complications, Mortality, Healthcare utilization, Primary hip arthroplasty, Primary knee arthroplasty

## Abstract

**Background:**

To assess whether Sjogren’s Syndrome (SS) is associated with outcomes after total knee or hip arthroplasty (TKA/THA).

**Methods:**

We used the 1998–2014 U.S. National Inpatient Sample data. We performed multivariable-adjusted logistic regression analyses to assess the association of SS with healthcare utilization (hospital charges, length of hospital stay, discharge to non-home setting), and in-hospital complications (implant infection, revision, transfusion, mortality), controlling for important covariates and confounders. In sensitivity analyses, we additionally adjusted the main models for hospital location/teaching status, bed size, and region**.**

**Results:**

We examined 4,116,485 primary THAs and 8,127,282 primary TKAs performed from 1998 to 2014; 12,772 (0.2%) primary TKAs and 6222 (0.2%) primary THAs were done in people with SS. In multivariable-adjusted models, SS was associated with a statistically significant higher odds ratio (OR; 95% confidence interval (CI)) of discharge to a rehabilitation/inpatient facility post-THA, 1.13 (1.00, 1.28), but not post-TKA, 0.93 (0.86, 1.02). We noted no differences in the length of hospital stay or hospital charges. SS was associated with significantly higher adjusted odds of in-hospital transfusion post-THA, 1.37 (1.22, 1.55) and post-TKA, 1.21 (1.10, 1.34). No significant differences by SS diagnosis were seen in hospital stay, hospital charges implant infection, implant revision or mortality rates.

**Conclusions:**

People with SS had higher transfusion rate post-TKA/THA, and higher rate of discharge to non-home setting post-THA. The lack of association of SS with post-arthroplasty complications should reassure patients, surgeons and policy-makers about the utility of TKA/THA in people with SS undergoing these procedures.

## Highlights

1. Sjogren’s Syndrome was associated with a significantly higher odds of discharge to a rehabilitation/inpatient facility post-THA, but not post-TKA.
2. Sjogren’s Syndrome was associated with significantly higher adjusted odds of in-hospital transfusion post-THA, and post-TKA.
3. Sjogren’s Syndrome was not associated with implant infection or revision or mortality post-THA/TKA.

## Background

Sjogren’s Syndrome (SS) is a systemic autoimmune disease, primarily of middle-aged women, characterized by dry eyes, dry mouth and systemic symptoms [1]. Its incidence and prevalence in the general population are 7/100,000 and 60/100,000 people, respectively [1]. SS can be primary, or secondary to diseases such as rheumatoid arthritis (RA), lupus, scleroderma, myositis etc. SS is associated with systemic inflammation with an over-expression of pro-inflammatory cytokines [2, 3].

With the aging of the population, an increasing number of people with SS and the general population are undergoing joint arthroplasty. Total knee arthroplasty (TKA) or hip arthroplasty (THA) are the two most common arthroplasty surgeries performed for end-stage arthritis [4]. Complications and healthcare utilization after arthroplasty are increased in other systemic inflammatory rheumatic disease such as RA, lupus and spondyloarthritis [5]. Whether SS, which is also a systemic autoimmune disease, has a similar impact on post-arthroplasty outcomes is unknown. Therefore, we assessed whether SS was associated with higher complication, mortality and healthcare utilization rates after primary TKA or THA.

## Methods

### Data source, study cohort and study outcomes

We used the 1998–2014 U.S. National Inpatient Sample (NIS) data, a 20% stratified sample of discharges from U.S. community hospitals [6]. NIS is the largest publicly available, de-identified all-payer inpatient health care database in the U.S. The University of Alabama at Birmingham’s Institutional Review Board approved this study and waived the need for informed consent (X120207004).

We identified a cohort of all hospitalizations with primary TKR or THA as the primary procedure by using validated International Classification of Disease, ninth revision, common modification (ICD-9-CM) codes for primary TKA (81.54) or primary THA (81.51) [7]. Among these, we identified those with and without Sjogren’s Syndrome (ICD-9-CM, 710.2), a valid approach with sensitivity of 95% and specificity of 96% [8], in non-primary position.

Our study outcomes of interest post-primary TKA/THA were: (1) three health care utilization measures: the discharge disposition to home vs. a rehabilitation/inpatient facility, the length of hospital stay above the median (> 3 days), and the hospital charges above the median for each calendar year; (2) three in-hospital complications identified by respective ICD-9-CM codes for transfusion, implant infection and implant revision; and (3) in-hospital mortality.

### Statistical analysis

We performed separate multivariable-adjusted logistic regression analyses for each outcome controlling for clinically important variables, some of which were also potential and/or known confounders of TKA/THA outcomes [9–13], including age, race, sex, income, underlying diagnosis (listed in the primary diagnosis position), Deyo-Charlson comorbidity index, and insurance payer. Deyo-Charlson comorbidity index [14], is a validated measure of medical comorbidity that includes 17 comorbidities with score ranging 0–25, higher score indicating more comorbidity load. Sensitivity analyses additionally adjusted for hospital bed size, location/teaching status, and region [15, 16]. Sensitivity analyses examined the main multivariable-adjusted logistic regression analyses in two SS subgroups: (1) Primary SS: no concomitant rheumatic disease diagnoses; (2) Secondary SS: presence of one or more concomitant rheumatic disease diagnoses (systemic lupus erythematosus, 710.0; systemic sclerosis, 710.1; sicca syndrome, 710.2; dermatomyositis, 710.3; polymyositis, 710.4; mixed connective tissue disease, 710.9; antiphospholipid syndrome, 289.81; rheumatoid arthritis, 714).

## Results

Of the 4,116,485 primary THAs and 8,127,282 primary TKAs performed from 1998 to 2014, 12,772 (0.2%) primary TKAs and 6222 (0.2%) primary THAs were done in people with SS. Compared to no SS, a higher proportion of those with a diagnosis of SS was female, had an underlying diagnosis of rheumatoid arthritis or a Deyo-Charlson index score of ≥2, for both primary THA and primary TKA cohorts (Table 1). Of these, the number with primary versus secondary SS were as follows: (1) Primary TKA: Primary SS, 8477 (66%); Secondary SS, 4295 (34%); (2) Primary THA: Primary SS, 4240 (68%); Secondary SS, 1982 (32%).

**Table 1:** Demographic and other cohort characteristics

	Primary TKA			Primary THA		
	Entire cohort	No Sjogren’s Syndrome	Sjogren’s Syndrome	Entire cohort	No Sjogren’s Syndrome	Sjogren’s Syndrome
**National Projections**^a^	*N* = 8,127,282^a^	N = 8,114,510^a^	*N* = 12,772^a^	N = 4,116,485^a^	*N* = 4,110,263^a^	*N* = 6222^a^
**Age, Mean (SE); median**	66.4 (0.03); 66.5	66.6 (0.02); 66.5	65.0 (0.20); 64.7	65.2 (0.04); 65.9	65.5 (0.04); 66.0	65.7 (0.32); 65.4
**Age category**						
< 50	430,141 (5.3%)	429,384 (5.3%)	757 (5.9%)	449,642 (10.9%)	449,170 (10.9%)	472 (7.6%)
50–64	2,872,619 (35.3%)	2,867,321 (35.3%)	5298 (41.5%)	1,364,821 (33.2%)	1,362,528 (33.1%)	2293 (36.9%)
65–79	3,969,942 (48.8%)	3,963,938 (48.8%)	6004 (47.0%)	1,732,015 (42.1%)	1,729,250 (42.1%)	2765 (44.4%)
≥ 80	850,131 (10.5%)	849,418 (10.5%)	713 (5.6%)	566,521 (13.8%)	565,828 (13.8%)	693 (11.1%)
**Gender**						
Female	5,126,809 (63.1%)	5,114,641 (63.0%)	12,168 (95.3%)	2,330,188 (56.6%)	2,324,441 (56.6%)	5747 (92.4%)
Male	2,985,796 (36.7%)	2,985,197 (36.8%)	599 (4.7%)	1,776,722 (43.2%)	1,776,247 (43.2%)	475 (7.6%)
**Race**						
White	5,507,281 (67.8%)	5,497,996 (67.8%)	9285 (72.7%)	2,882,041 (70.0%)	2,877,245 (70.0%)	4796 (77.1%)
Black	472,393 (5.8%)	471,784 (5.8%)	609 (4.8%)	225,772 (5.5%)	225,579 (5.5%)	193 (3.1%)
Hispanic	340,292 (4.2%)	339,802 (4.2%)	490 (3.8%)	104,384 (2.5%)	104,234 (2.5%)	150 (2.4%)
Other/Missing	1,807,101 (22.2%)	1,804,713 (22.2%)	2388 (18.7%)	904,234 (22.0%)	903,151 (22.0%)	1083 (17.4%)
**Deyo-Charlson Score**						
0	4,104,090 (50.5%)	4,100,452 (50.5%)	3638 (28.5%)	2,193,574 (53.3%)	2,191,576 (53.3%)	1998 (32.1%)
1	2,064,888 (25.4%)	2,060,861 (25.4%)	4027 (31.5%)	926,286 (22.5%)	924,372 (22.5%)	1914 (30.8%)
≥ 2	1,958,305 (24.1%)	1,953,198 (24.1%)	5107 (40.0%)	996,623 (24.2%)	994,314 (24.2%)	2309 (37.1%)
**Primary Diagnosis**						
Rheumatoid arthritis	64,126 (0.8%)	63,422 (0.8%)	704 (5.5%)	29,173 (0.7%)	28,938 (0.7%)	235 (3.8%)
Aseptic bone necrosis	21,031 (0.3%)	20,963 (0.3%)	68 (0.5%)	285,623 (6.9%)	284,864 (6.9%)	759 (12.2%)
Osteoarthritis	7,866,436 (96.8%)	7,854,625 (96.8%)	11,811 (92.5%)	3,447,224 (83.7%)	3,442,590 (83.8%)	4634 (74.5%)
Other	173,672 (2.1%)	173,488 (2.1%)	184 (1.4%)	354,307 (8.6%)	353,713 (8.6%)	594 (9.5%)
Fracture	1904 (0.0%)	1904 (0.0%)	0 (0.0%)	117 (0.0%)	117 (0.0%)	0 (0.0%)
**Hospital Location/Teaching**						
Rural	1,041,159 (12.8%)	1,040,000 (12.8%)	1159 (9.1%)	444,188 (10.8%)	443,736 (10.8%)	452 (7.3%)
Urban	3,665,766 (45.1%)	3,660,404 (45.1%)	5362 (42.0%)	1,722,391 (41.8%)	1,719,952 (41.8%)	2439 (39.2%)
Urban Teaching	3,398,114 (41.8%)	3,391,899 (41.8%)	6215 (48.7%)	1,939,989 (47.1%)	1,936,692 (47.1%)	3297 (53.0%)
**Insurance**						
Medicaid	226,917 (2.8%)	226,680 (2.8%)	237 (1.9%)	138,809 (3.4%)	138,668 (3.4%)	141 (2.3%)
Medicare	4,631,192 (57.0%)	4,623,773 (57.0%)	7419 (58.1%)	2,234,674 (54.3%)	2,230,839 (54.3%)	3835 (61.6%)
Other	267,624 (3.3%)	267,297 (3.3%)	327 (2.6%)	102,277 (2.5%)	102,205 (2.5%)	72 (1.2%)
Private	2,947,676 (36.3%)	2,942,954 (36.3%)	4722 (37.0%)	1,600,830 (38.9%)	1,598,692 (38.9%)	2138 (34.4%)
Self	36,589 (0.5%)	36,535 (0.5%)	54 (0.4%)	32,307 (0.8%)	32,288 (0.8%)	19 (0.3%)
**Income Category**						
0-25th percentile	1,511,693 (18.6%)	1,509,616 (18.6%)	2077 (16.3%)	653,243 (15.9%)	652,323 (15.9%)	920 (14.8%)
25-50th percentile	2,156,222 (26.5%)	2,153,260 (26.5%)	2962 (23.2%)	1,009,677 (24.5%)	1,008,178 (24.5%)	1499 (24.1%)
50-75th percentile	2,158,012 (26.6%)	2,154,363 (26.5%)	3649 (28.6%)	1,086,953 (26.4%)	1,085,319 (26.4%)	1634 (26.3%)
75-100th percentile	2,151,430 (26.5%)	2,147,588 (26.5%)	3842 (30.1%)	1,285,854 (31.2%)	1,283,805 (31.2%)	2049 (32.9%)
**Hospital Bed size**						
Small	1,408,128 (17.3%)	1,405,774 (17.3%)	2354 (18.4%)	685,209 (16.6%)	684,163 (16.6%)	1046 (16.8%)
Medium	2,107,249 (25.9%)	2,103,919 (25.9%)	3330 (26.1%)	1,037,561 (25.2%)	1,036,014 (25.2%)	1547 (24.9%)
Large	4,589,661 (56.5%)	4,582,609 (56.5%)	7052 (55.2%)	2,383,797 (57.9%)	2,380,202 (57.9%)	3595 (57.8%)
**Hospital Region**						
Northeast	1,359,230 (16.7%)	1,357,194 (16.7%)	2036 (15.9%)	818,699 (19.9%)	817,401 (19.9%)	1298 (20.9%)
Midwest	2,269,799 (27.9%)	2,266,484 (27.9%)	3315 (26.0%)	1,089,883 (26.5%)	1,088,481 (26.5%)	1402 (22.5%)
South	2,957,629 (36.4%)	2,953,077 (36.4%)	4552 (35.6%)	1,358,856 (33.0%)	1,356,707 (33.0%)	2149 (34.5%)
West	1,540,624 (19.0%)	1,537,755 (19.0%)	2869 (22.5%)	849,045 (20.6%)	847,673 (20.6%)	1372 (22.1%)
**In-hospital Complications and Healthcare Utilization**						
**Transfusion**	1,288,545 (15.9%)	1,286,034 (15.8%)	2511 (19.7%)	937,802 (22.8%)	935,821 (22.8%)	1981 (31.8%)
**Implant Infection**	8165 (0.1%)	8151 (0.1%)	14 (0.1%)	7592 (0.2%)	7573 (0.2%)	19 (0.3%)
**Implant Revision**	15,310 (0.2%)	15,281 (0.2%)	29 (0.2%)	17,931 (0.4%)	17,884 (0.4%)	47 (0.8%)
**Death**	7875 (0.1%)	7865 (0.1%)	10 (0.1%)	8889 (0.2%)	8880 (0.2%)	9 (0.2%)
**Discharge Status**						
Home	4,965,279 (61.1%)	4,957,619 (61.1%)	7660 (60.0%)	2,448,107 (59.5%)	2,444,859 (59.5%)	3248 (52.2%)
Rehabilitation/inpatient facility	3,130,288 (38.5%)	3,125,196 (38.5%)	5092 (39.9%)	1,649,103 (40.1%)	1,646,152 (40.0%)	2951 (47.4%)
**Hospital stay in days: Mean (SE); median**	3.53 (0.01); 2.70	3.54 (0.01); 2.70	3.48 (0.03); 2.68	3.71 (0.01); 2.74	3.73 (0.01); 2.75	3.76 (0.06); 2.77
**Hospital stay in days**						
≤ 3	5,218,769 (64.2%)	5,210,306 (64.2%)	8463 (66.3%)	2,499,883 (60.7%)	2,496,141 (60.7%)	3742 (60.1%)
> 3	2,908,513 (35.8%)	2,904,204 (35.8%)	4309 (33.7%)	1,616,602 (39.3%)	1,614,122 (39.3%)	2480 (39.9%)
**Total Hospital Charges, in U.S. $, Mean (SE); Median**	42,336 (235); 35,891	42,441 (236); 35,946	46,429 (646); 39,793	44,635 (268); 37,658	44,859 (270); 37,785	50,084 (980); 42,495

^a^U.S. National estimates were based on the following actual counts in the NIS, which is a 20% sample of all U.S. hospitalizations (https://www.hcup-us.ahrq.gov/nisoverview.jsp): **TKA,***N* = 1,690,531; No Sjogren’s syndrome, N = 1,687,895; Sjogren’s syndrome, *N* = 2636; **THA**, *N* = 855,634; No Sjogren’s syndrome, *N* = 854,353; Sjogren’s syndrome, *N* = 1281; N (%), unless specified otherwise

Overall, 12,772 (0.2%) of people with TKA hospitalizations had Sjogren’s Syndrome; 6222 (0.2%) of people with THA hospitalizations had Sjogren’s Syndrome

SS was associated with a statistically significant higher odds ratio (OR; 95% confidence interval (CI)) of discharge to a rehabilitation/inpatient facility post-THA, 1.13 (1.00, 1.28), but not post-TKA, 0.93 (0.86, 1.02) after multivariable-adjustment (Table 2). We noted no differences in the length of hospital stay or hospital charges. We found significantly higher adjusted odds of in-hospital transfusion post-THA, 1.37 (1.22, 1.55) and post-TKA, 1.21 (1.10, 1.34) associated with SS (Table 2). No differences by SS diagnosis were seen in implant infection, implant revision or mortality rates. Sensitivity analyses adjusting for additional hospital characteristics confirmed the findings from the main analyses with minimal attenuation of ORs (Table 2).

**Table 2:** Multivariable-adjusted association of Sjogren’s syndrome with complications and healthcare utilization outcomes after primary THA or primary TKA in the main model and the sensitivity analyses

	Primary TKA		Primary THA	
	Main Model^a^	Sensitivity analyses^b^	Main Model^a^	Sensitivity analyses^b^
	Odds Ratio (95% CI)	Odds Ratio (95% CI)	Odds Ratio (95% CI)	Odds Ratio (95% CI)
Discharge to a rehabilitation/inpatient facility	0.93 (0.86, 1.02)	0.97 (0.89, 1.06)	**1.13 (1.00, 1.28)**	**1.14 (1.01, 1.30)**
Length of hospital stay > 3 days ^c^	0.96 (0.88, 1.04)	0.97 (0.89, 1.05)	0.99 (0.88, 1.12)	1.00 (0.89, 1.12)
Total hospital charge above the median ^d^	0.98 (0.91,1.06)	0.94 (0.87,1.01)	1.09 (0.98,1.22)	1.05 (0.93,1.17)
In-hospital complications				
Transfusion	**1.21 (1.10, 1.34)**	**1.23 (1.12, 1.36)**	**1.37 (1.22, 1.55)**	**1.37 (1.21, 1.55)**
Infection	1.22 (0.39, 3.78)	1.21 (0.39, 3.78)	1.32 (0.44, 4.01)	1.32 (0.44, 3.92)
Revision	1.41 (0.63, 3.15)	1.39 (0.62, 3.11)	1.72 (0.80, 3.72)	1.73 (0.79, 3.76)
Death	0.98 (0.24, 3.93)	0.99 (0.25, 3.97)	0.85 (0.43, 1.70)	0.86 (0.42, 1.76)

^a^ Main model was adjusted for socio-demographics (age, race/ethnicity, gender, income), Deyo-Charlson comorbidity index, insurance payer and the underlying diagnosis for primary TKA or primary THA, respectively

^b^ Sensitivity analyses adjusted each main model additionally for hospital characteristics including hospital location/teaching status, hospital region, and hospital bed size

^c^ The median length of hospital stay was 2.7 days for TKA and 2.8 days for THA, both rounded off to 3 days

^d^ Median hospital charges were provided for each calendar year by the NIS:

Primary THA: 1998, $19,717; 1999, $20,514; 2000, $22,333; 2001, $24,189; 2002, $26,729; 2003, $29,858; 2004, $32,607; 2005, $34,615; 2006, $36,164; 2007, $39,675; 2008, $43,064; 2009, $41,602; 2010, $45,186; 2011, $48,898; 2012, $48,927; 2013, $50,827; 2014, $51,482

Primary TKA: 1998, $18,987; 1999, $19,717; 2000, $21,043; 2001, $22,622; 2002, $24,736; 2003, $26,696; 2004, $29,535; 2005, $31,686; 2006, $34,254;2007, $36,356; 2008, $38,920; 2009, $39,681; 2010, $42,191; 2011, $45,616; 2012, $46,438; 2013, $48,248; 2014, $49,330

**BOLD indicates significant odds ratio**

*CI* confidence interval

Sensitivity analyses were performed for primary vs. secondary SS (Additional file 1). Main study findings were reproduced for the higher odds of transfusion for both primary and secondary SS. Association of SS with discharge to a rehabilitation/inpatient facility post-THA were no longer significant, when subgroups of primary and secondary SS were examined. Additionally, primary SS was associated with higher odds of revision post-TKA; and secondary SS with higher odds of length of hospital stay > 3 days and lower odds of death post-THA (Additional file 1).

## Discussion

In a nationally representative U.S. sample, we found that SS, a multi-system autoimmune disease, was associated with a significantly higher odds of discharge to a rehabilitation/inpatient facility post-THA with 1.13-fold higher odds, but not post-TKA. Fatigue [17] and reduced functional ability [18], frequent in SS, may interfere with optimal in-hospital rehabilitation and recovery. This can potentially increase the risk of discharge to a rehabilitation or an inpatient facility. The reason for increased risk of discharge to a rehabilitation facility only after THA, but not TKA, may warrant further study. The strength of association was not very high, therefore the absolute impact of this significant association in people with SS may be small.

SS was associated with a higher risk of transfusion after THA and TKA, at 1.2–1.4 fold higher. This association was reproduced in subgroups of people with primary or secondary SS. Systemic inflammation [2, 3] in SS with associated anemia [19] and cytopenia [17] can lead to a higher transfusion risk post-THA/TKA.

We found that SS was not associated with complications including implant infection, revision or mortality after primary THA or TKA. Our finding contrasts with previously noted higher post-TKA/THA complication rates in people with other systemic inflammatory conditions, such as RA, SpA or lupus [5]. Limited joint or organ involvement in SS compared to RA, SpA or lupus [20] and/or the assessment of in-hospital complications in our study (vs. all post-operative complications) may explain these differences. An absence of an association of SS with post-arthroplasty infection, revision or mortality should reassure patients with SS and surgeons that these risks are not increased post-arthroplasty. The implications of possibly increased infection in primary SS post-TKA and decreased mortality in secondary SS post-THA are unclear since these were sub-group analyses; we believe that these findings need further study and replication.

Study limitations include residual confounding bias (direction of bias unclear), the lack of data on SS severity and laboratory measures, misclassification bias which likely biased findings towards the null, and the lack of longitudinal data. Bilateral simultaneous THA/TKA can not be distinguished from unilateral procedures in the NIS; however, these constitute < 1% THA and < 3% TKA, and therefore the bias is likely small. We were unable to separately examine the associations for primary versus secondary Sjogren’s syndrome due to the lack of a separate ICD-9 code for these. However, since the analyses are adjusted for underlying reason for THA/TKA which includes all rheumatic conditions associated with THA/TKA, the associations are for the presence versus absence of Sjogren’s Syndrome. These findings should alert the clinician to a higher transfusion risk in SS patients undergoing THA or TKA, and reassure the policy-makers that early THA/TKA outcomes are only minimally impacted by SS.

## Conclusions

In conclusion, SS was associated with a higher risk of transfusion after THA and TKA, higher odds of discharge to a rehabilitation/inpatient facility post-THA, but not post-TKA. SS was not associated with post-arthroplasty infection, revision or mortality. These findings can inform patients, providers and policy-makers regarding the minimal impact of SS on post-primary TKA/THA outcomes.

## Supplementary information

**Additional file 1.** Multivariable-adjusted association of Primary versus Secondary Sjogren’s syndrome (SS) with complications and healthcare utilization outcomes after primary THA or primary TKA in the main model.

## Data Availability

These data are easily available from the Agency for Healthcare Research and Quality (AHRQ’s) “Healthcare Cost and Utilization Project (HCUP)” and can be obtained after completing an on-line Data Use Agreement training session and signing a Data Use Agreement. The contact information for requesting the data is as follows: HCUP Central Distributor. Phone: (866) 556–4287 (toll-free). Fax: (866) 792–5313. E-mail: HCUPDistributor@ahrq.gov

## Abbreviations

SS: Sjogren’s Syndrome
NIS: National Inpatient Sample
TKA: Total knee arthroplasty
THA: Total hip arthroplasty
OR: Odds ratio
CI: Confidence interval
SE: Standard error
ICD-9-CM: International Classification of Diseases, Ninth Revision, Clinical Modification
