# Supplementary material for: Sjogren’s syndrome is associated with higher rate of non-home discharge after primary hip arthroplasty and higher transfusion rates after primary hip or knee arthroplasty: a U.S. cohort study
Source: BMC Musculoskelet Disord. 2020 Jul 25;21:492. doi: 10.1186/s12891-020-03514-9 (PMC7382828; doi:10.1186/s12891-020-03514-9)
Supplement: Supplementary file 1 — Additional file 1. Multivariable-adjusted association of Primary versus Secondary Sjogren’s syndrome (SS) with complications and healthcare utilization outcomes after primary THA or primary TKA in the main model. [file 12891_2020_3514_MOESM1_ESM.docx]

**Appendix 1.** Multivariable-adjusted association of **Primary versus Secondary Sjogren’s syndrome** (SS) with complications and healthcare utilization outcomes after primary THA or primary TKA in the main model

|  | **Primary SS** | | **Secondary SS** | |
| --- | --- | --- | --- | --- |
|  | **Primary TKA** | **Primary THA** | **Primary TKA** | **Primary THA** |
|  | Main Model | Main Model | Main Model | Main Model |
|  | Odds Ratio (95% CI) | Odds Ratio (95% CI) | Odds Ratio (95% CI) | Odds Ratio (95% CI) |
|  |  |  |  |  |
| Discharge to a rehabilitation/inpatient facility | 0.94 (0.85, 1.04) | 1.10 (0.95, 1.28) | 0.92 (0.79, 1.06) | 1.18 (0.95, 1.47) |
| Length of hospital stay >3 days ^3^ | 0.91 (0.82, 1.01) | 0.88 (0.76, 1.02) | 1.06 (0.92, 1.23) | **1.28 (1.04, 1.57)** |
| Total hospital charge above the median ^4^ | 0.91 (0.71, 1.16) | 1.09 (0.74, 1.59) | 1.25 (0.86, 1.82) | 1.14 (0.66, 2.00) |
|  |  |  |  |  |
| In-hospital complications |  |  |  |  |
| Transfusion | 1.12 (0.99, 1.26) | **1.24 (1.07, 1.44)** | **1.42 (1.21, 1.68)** | **1.70 (1.38, 2.09)** |
| Infection | 1.87 (0.60, 5.81) | 1.47 (0.37, 5.82) | Not applicable* | 1.06 (0.18, 6.17) |
| Revision | **2.29 (1.03, 5.10)** | 1.58 (0.57, 4.42) | Not applicable* | 2.06 (0.68, 6.22) |
| Death | 1.57 (0.39, 6.30) | 0.98 (0.40, 2.41) | Not applicable* | **0.62 (0.51, 0.76)** |
| *Not applicable due to the lack of enough events to obtain an estimate | | | | |
